# Investigation of factors affecting prediction of protein-protein interaction networks by phylogenetic profiling

**DOI:** 10.1186/1471-2164-8-393

**Published:** 2007-10-29

**Authors:** Anis Karimpour-Fard, Lawrence Hunter, Ryan T Gill

**Affiliations:** 1 Center for Computational Pharmacology, University of Colorado School of Medicine, Aurora, Colorado 80045, USA; 2 Department of Chemical and Biological Engineering, University of Colorado, Boulder, CO 80309, USA

## Abstract

**Background:**

The use of computational methods for predicting protein interaction networks will continue to grow with the number of fully sequenced genomes available. The Co-Conservation method, also known as the Phylogenetic profiles method, is a well-established computational tool for predicting functional relationships between proteins.

**Results:**

Here, we examined how various aspects of this method affect the accuracy and topology of protein interaction networks. We have shown that the choice of reference genome influences the number of predictions involving proteins of previously unknown function, the accuracy of predicted interactions, and the topology of predicted interaction networks. We show that while such results are relatively insensitive to the *E*-value threshold used in defining homologs, predicted interactions are influenced by the similarity metric that is employed. We show that differences in predicted protein interactions are biologically meaningful, where judicious selection of reference genomes, or use of a new scoring scheme that explicitly considers reference genome relatedness, produces known protein interactions as well as predicted protein interactions involving coordinated biological processes that are not accessible using currently available databases.

**Conclusion:**

These studies should prove valuable for future studies seeking to further improve phylogenetic profiling methodologies as well for efforts to efficiently employ such methods to develop new biological insights.

## Background

Genome sequencing projects are rapidly increasing the raw data available for predicting protein function and protein interaction networks. The best established method for function prediction is based on sequence homology to proteins of known function. Unfortunately, strictly homology-based predictions are of limited use due to the large number of homologous protein families with no known function for any single member [1-3]. An alternative method for predicting protein function is the Phylogenetic profile method, also known as the Co-Conservation method, which rests on the premise that functionally related proteins are gained or lost together over the course of evolution [4]. This method predicts functional interactions between pairs of proteins in a target organism by determining whether both proteins are consistently present or absent across a set of reference genomes. These protein-protein interactions (PPI) are distinct from physical interactions as they capture putative functional relationships. Sequence similarity is used only to identify homologs, not to infer function. Since first introduced by Pellegrini *et al. *[4], Phylogenetic profiling has been successfully applied to the prediction of protein function by several groups and demonstrated to be more powerful than sequence similarity alone at predicting protein function [5-11].

Currently several web-based databases compile predictions of protein-protein interactions (PPIs), e.g. PLEX [7], String [5] and Prolinks [8]. These databases either use all available bacterial genomes at the time of implementation or a select subset of bacterial genomes without focusing on how the selection of the bacteria will influence the PPIs. Several groups have attempted to address this issue, including a number of methods that account for genome phylogeny when scoring profile similarities [12,13]. Barker *et al. *applied maximum likelihood statistical modeling for predicting functional protein linkages based on Phylogenetic profiling [13]. Their method detected independent instances of the correlated gain or loss of protein pairs on phylogenetic trees, reducing the high rates of false positives observed in conventional across-species methods that do not explicitly incorporate a phylogeny [13]. Jothi *et al*. did a study using 16 different reference sets of genomes, using combinations of bacterial, archaea and eukaryotic genomes. They showed using a combination of bacterial and archaea genomes as a reference set could be enough to make accurate functional linkage predictions [14]. Cokus *et al*. found phylogenetic relationships between genomes by using the first order of the genomes within profiles and then enumerating runs of consecutive matches to compute the accuracy of the probability of observing these phylogenetic relationships [15]. Zheng *et al. *constructed Phylogenetic profiles based upon the presence or absence of neighboring protein pairs within a genome [16]. They demonstrated that the inclusion of more genomes (68 vs. 30) resulted in better performance for PPI predictions, however, they did not provide a strategy for bacteria selection. Sun *et al*. showed that accuracy of PPI predictions can be improved by using a set of genomes which are maximally distinct from one another [17]. We have noted the same phenomenon here, but further show that selection of groups of bacteria that are closely related either phenotypically or genotypically generates biologically relevant information that is missed when other methods for grouping bacteria are employed.

It is sensible that inclusion of genomes from organisms that exist in similar environmental niches (i.e. rhizosphere bacteria), share certain phenotypic properties (i.e. motility), or that are from the same species (i.e. different strains of E. coli) might bias protein interaction network predictions in an undesirable manner. The challenge is that the extent of such biases remains uncharacterized, and thus methods for guiding the selection of relevant reference genomes are lacking. Here, we have examined such biases and then used our studies to developed a new scoring scheme to provide guidance for the selection of reference genomes in Phylogenetic profiling efforts.

## Results

Phylogenetic profiling methods work by i) creating a Phylogenetic profile vector where P_ij _= 1 indicates a homolog exists between protein i in the target genome and a protein in a reference genome j, ii) calculating similarity measurements on the profile vectors for each pair of genes in the target genome, and iii) defining protein interactions in the target genome based on proteins sharing a profile similarity value greater than a threshold value. Using *E. coli *K12 as the target genome, we have evaluated how changing different aspects of this process, including the use of a new metric for defining similarity, affect predicted protein interaction networks.

### Comparison and evaluation of protein-protein interaction

#### The effect of reference genome selection on interactions with proteins of unknown function

**Figure 1:**
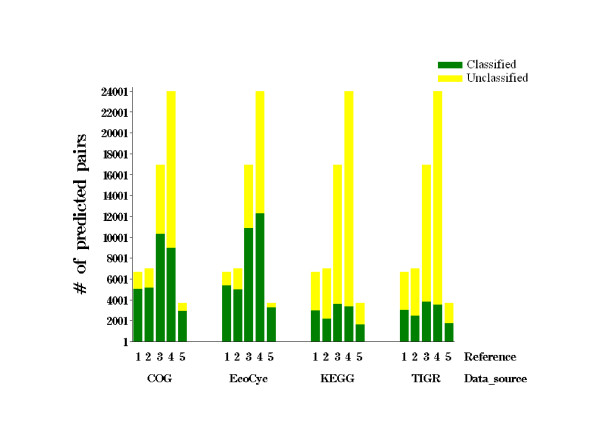
Comparison of unclassified proteins in published databases. The reference sets are **1 – All **bacteria using *Inverse homology ***2 – All **bacteria; **3 – Motile **bacteria; **4 – Protebacteria**; **5 – Selected **bacteria. Pearson correlation coefficient on binary profiles was used to measure profile similarity for reference two through five. For Reference **1**, Pearson correlation coefficient applied to *Inverse homology*. Pearson correlation coefficient with confidence r > 0.8 *E*-value 10^-5 ^was used as threshold for BLAST. Classified (green); unclassified (yellow).

Many function prediction methods rely on the assumption that functionally related proteins interact [11,18-21]. Thus, the utility of an interaction network whether based on physical, genetic, or computationally predicted interactions can be assessed by determining the number of predicted interactions involving proteins that have unclassified functions in publicly accessible databases that rely upon homology based methods for functional assignment (Figure 1). For each network constructed using four sets of reference genomes (**All **(268 bacteria), **Selected **(75 bacteria), **Proteobacteria **(130 bacteria), and **Motile **(104)), we identified the total number of PPI pairs that have at least one of the proteins labeled as unclassified. Our results showed the number of unclassified proteins was greatest when the reference genomes were either closely related phylogenetically (**Proteobacteria**) or phenotypically (**Motile**), regardless of the database used for assigning function. The **Selected **set of reference genomes contains only a single representative strain from bacterial species where more than one strain has been fully sequenced (i.e. *E. coli*). As such, this set of reference genomes can be considered the least closely related of all of the reference sets examined. Protein interaction networks created from the **Selected **set consistently contained the least number of proteins pairs involving with unknown function. It is of note that the EcoCyc [22] and Cluster of Orthologous Groups (COG) databases contained the least number of unclassified proteins, and therefore were chosen for the additional studies described next.

#### Comparison of different combinations of reference genomes and E-value thresholds

**Figure 2:**
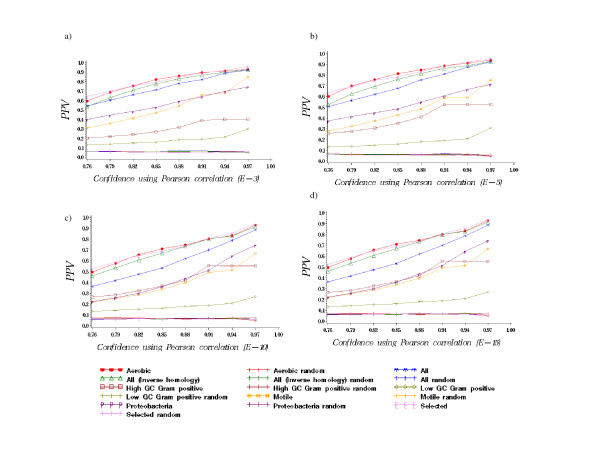
Comparison of different combinations of reference genome sets and *E*-value thresholds. a) E^-3 ^b) E^-5 ^c) E^-10 ^d) E^-15^. Positive Predicted Value (PPV) was calculated using COG functional category as described in the methods section. Random indicates protein-protein interactions generated by scrambling proteins identities for each reference genome set. In Additional file 1, PPV was calculated using EcoCyc.

The threshold value used to identify homologs between target and reference genomes is a key measure when assessing the effect of reference genomes on protein interaction networks. If the threshold value is low, then many homologs may be identified even among distantly related bacterial species. To assess this concern, we evaluated the Positive Predicted Value (PPV = TP/(TP+FP)) for seven sets of reference genomes (**All**, **Selected**, **Proteobacteria**, **Low GC **Gram positive bacteria, **High GC **Gram positive bacteria, **Motile**, and **Aerobic**) for each of four different *E*-value thresholds across a range of similarity (confidence) requirements using EcoCyc and COG functional categories (Figure 2 and Additional file 1). True positives (TP) correspond to interactions where the proteins pair share the same function (COG) or appear in the same complex, pathway, operon, or paralogous group (EcoCyc) or are homologs. All other interactions were considered false positives (FP). The significance of interactions between proteins with shared COG function was also evaluated using randomized data. To preserve the distribution of function assignment and network topology while disrupting the correlation among function assignments, the proteins identities were scrambled at random and the profile similarities were calculated. Our results indicated that the PPV for all random sets were significantly different than when using the original data (p-value < 0.0001) (Figure 2).

The *E*-value chosen for non-randomized interactions did not substantially alter the trends observed among the different reference genomes except for **High GC **gram positive bacteria. This observation is likely explained by the relatively low number of genomes contained within the **High GC **Gram positive reference set (22 genomes). PPV was the best for **Selected**, **All **(*inverse homology*) and **Aerobic**, indicating that these reference genome sets interactions commonly occurred between proteins of the same function and was worst for **Proteobacteria**, **Motile**, and **Low GC **reference genome sets, with the **All **and **High GC **groups always remaining in between these two other groups. At restrictive *E*-value thresholds (E < 10^-15^), this result reinforced those reported above stating that reference genomes of maximum diversity created the most accurate (highest PPV) protein interaction networks. What was surprising was that these trends were observed even at less restrictive *E*-value thresholds, where it might be expected that proteins of lower similarity, and thus more likely to be false positives, would presumably be added to the network. In fact, the number of Co-Conserved proteins was similar regardless of the *E*-value threshold for all reference genomes (see Tables 1, 2). Thus, even if the number of predicted interaction pairs was altered (which is also a function of Co-Conserved vector similarity), the number of new proteins added to the overall network was relatively constant. These observations suggest that using reference genomes of increased relatedness produces less accurate results (lower PPV) because the predicted networks either 1) include proteins from different function classifications that truly act together in larger coordinated process (and thus are true positives not false positives) or 2) include proteins where at least one protein is poorly characterized. Moreover, these observations suggest that methods for assessing similarity might be improved by explicitly considering the relatedness of reference genomes.

**Table 1:** Predicted protein-protein interaction pairs using different sets of reference genomes.

Reference genome	No. clusters >2	No. Co-Conserved predicted pairs	No. Co-Conserved proteins
**Aerobic**	112	3,825	1,410
**All**	143	6,987	1,700
**All **(*Inverse homology*)	146	6,694	1,770
**High GC Gram positive**	33	7,932	1,389
**Low GC Gram negative**	43	56,659	1,667
**Motile**	110	16,905	1,990
**Proteobacteria**	111	24,047	2,072
**Selected**	133	7,408	1,361

Pearson correlation coefficient with confidence 0.8 and *E*-value of 10^-5 ^were used on binary profile vectors unless noted.

**Table 2:** Predicted protein-protein interaction pairs using different set of reference genomes.

Reference genome	No. clusters >2	No. Co-Conserved predicted pairs	No. Co-Conserved proteins
**Aerobic**	105	3,890	1,233
**All**	122	9,539	1,539
**All **(*Inverse homology*)	129	6,469	1,557
**High GC Gram positive**	27	4,237	1,058
**Low GC Gram positive**	33	34,968	1,325
**Motile**	88	24,198	1,972
**Proteobacteria**	101	48,730	2,119
**Selected**	109	3,839	1,206

Pearson correlation coefficient with confidence 0.8 and *E*-value of 10^-15 ^were used on binary profile vectors unless noted.

#### Network topology using different reference genome

The topology of bacterial Co-Conservation networks exhibited interesting similarities and differences as a function of reference genomes. The clustering coefficient is the edge density in the neighbors of a protein [23]. The average clustering coefficient of the network across all reference genome sets was high (Table 3), indicating that there was a short path between any two proteins in a cluster, and that proteins tended to be Co-Conserved in highly connected groups. Interestingly, different reference sets showed a large variation in the average degree of connectivity (Table 3). The **Low GC **reference set had the highest average connectivity while the **Aerobic **and **Selected **reference sets had the lowest average connectivity (Table 3). Together these results show that networks based on **Aerobic **or **Selected **reference sets, which are maximally distinct either phenotypically or genotypically generate many small, dense clusters. Interestingly, the result in Figure 2 for **Aerobic **and **Selected **suggest these small clusters are functionally homogenous, since high PPV values indicate many interactions among proteins of the same function.

**Table 3:** Topological analysis network measured using different sets of reference genomes.

Reference	Average clustering coefficient	Average connectivity	No. Co-Conserved proteins
**Aerobic**	0.77	5.42	1,410
**All**	0.81	8.22	1,700
**All **(*inverse homology*)	0.78	7.56	1,770
**High GC**	0.63	11.42	1,389
**Low GC**	0.72	67.97	1,667
**Motile**	0.74	16.98	1,990
**Proteobacteria**	0.75	23.21	2,072
**Selected**	0.82	5.44	1,360

Pearson correlation coefficient with confidence 0.8 and *E*-value of 10^-5 ^were used on binary profile vectors.

#### Comparison of different scoring schemes

**Figure 3:**
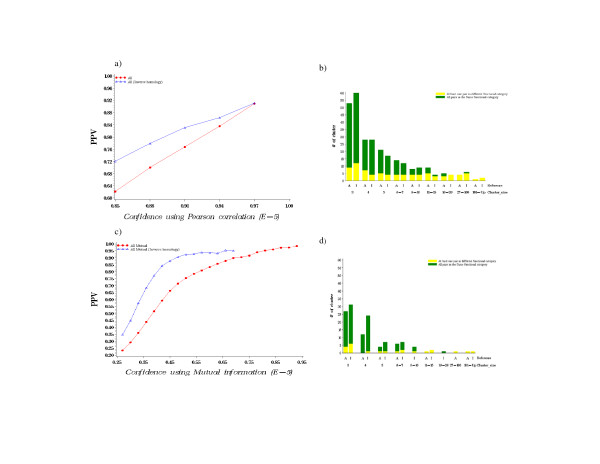
Pearson correlation coefficient and Mutual Information applied to *Inverse homology*. The PPV was calculated using COG functional categories: a) the PPV before and after *Inverse homology *applied to Pearson correlation coefficient and show topology of the network using **All **as reference before and after applying *Inverse homology (*confidence r > 0.8 and *E*-value 10^-5^); b) the PPV before and after *Inverse homology *applied to Mutual Information and the topology of the network using **All **as reference before and after applying *Inverse homology (*confidence MI > 0.3 and *E*-value 10^-5^).

Pearson correlation and Mutual Information are two common methods for measuring vector similarity (see Methods). Previous Phylogenetic profiling methods have either used protein vectors comprised of binary values (i.e. homolog = 1, no homolog = 0) [4] or vectors normalized with *E*-values [7,24]. We wanted to employ a vector weighting metric that considered how related the target and reference genomes were for identified homologs. To this end, we developed the *Inverse homology *scoring scheme, which weights each protein vector value fractionally by the inverse of the number of homologs between the target and reference genomes as a fraction of the total number of proteins in the reference genome. This metric emphasizes homologs identified in distantly related genomes relative to those identified in more closely related genomes. To evaluate this method of weighing, we applied the Mutual Information, Pearson correlation coefficient and *Inverse homology *scoring schemes to the **All **(i.e. 268 bacteria) reference genome set and assessed predicted protein interactions (PPV) (see Figure 3). Our results indicate that the *Inverse homology *scoring scheme increases PPV across a range of confidence values when Pearson correlation is used as the similarity metric (Figure 3a). Note that this trend was also observed over a range of different *E*-value thresholds as presented in Figure 2. This same trend was observed when Mutual Information was employed (Figure 3c). In contrast, the topology of networks created using Pearson correlation was similar regardless of whether or not *Inverse homology *was employed but was different in the case of Mutual Information (see Figure 3b and 3d). Specifically, the use of the *Inverse homology *weighting criteria decreased connectivity and the number of clusters containing less than ten proteins, thus altering the topology of the predicted protein interaction network.

### Selection of reference genome affects protein-protein interaction predictions

**Figure 4:**
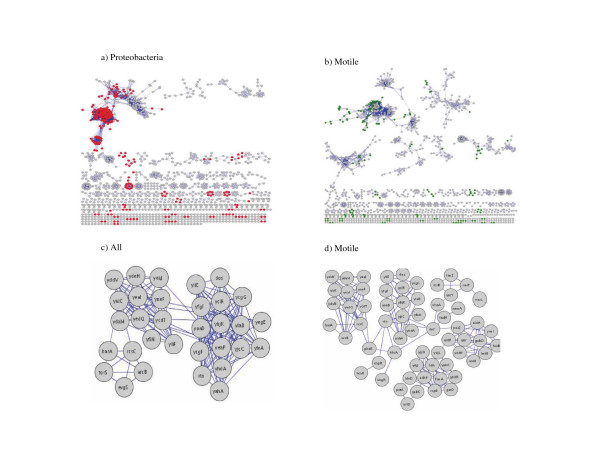
Selection of reference genome affects protein-protein interaction predictions.a) PPIs generated using **Proteobacteria **as the reference genome set. Red nodes are the nodes that are predicted when the reference genome is **Proteobacteria **and not predicted when the reference genome is **All **(confidence for **Proteobacteria **is r > 0.9 and for **All **is r > 0.8 since these two confidences give similiar PPV). b) PPIs generated using **Motile **as the reference genome. Green nodes are the nodes that are predicted when the reference genome is **Motile **and not predicted when the reference genome is **All **(confidence for **Motile **is r > 0.88 and for **All **is r > 0.8 since these two confidences give the same PPV) c) Two of the clusters that is predicted when the reference genome are **Protebacteria **not **All**. The proteins within this cluster are mainly membrane proteins that are Co-Conserved with hypothetical or unclassified proteins. d) Comparison of GGDEF clusters with two different type of reference selection. (**All **and **Motile**). When **All **is used as the reference genome set, the GGDEF domain is Co-Conserved with EAL domain and two component regulatory system proteins but when **Motile **is used as a reference genome set, GGDEF domain is Co-Conserved with EAL domain, two component regulatory system, regulator and proteins involved in metabolism. All these processes are known to contribute to the common phenomenon of biofilm formation.

Our analysis of *E. coli *K12 as a target genome indicated that the selection of reference organisms had a substantial effect on the overall properties of predicted protein interaction networks. An important additional issue was the details of how such effects were manifested at the level of specific protein-protein interactions and protein interaction clusters. To investigate this issue, we assessed the extent to which unique protein interaction clusters were predicted when different reference genomes were employed (Figure 4). We show representative results of such comparisons for **Proteobacteria **versus **All **(Figure 4a) or **Motile **versus **All **(Figure 4b) reference genomes. Many protein nodes within interaction clusters were uniquely identified when either **Proteobacteria **or **Motile **reference genomes sub-sets were used. These clusters ranged in size from as few as two proteins to some of the largest protein interaction clusters, clearly indicating the importance of reference genome selection on protein interaction predictions. The key question here is whether or not such unique interactions are biologically insightful.

Figure 4c–d show biofilm related *E. coli *K12 protein interaction networks developed when either **All **or **Motile **reference genome sets were used. When **All **was used as the reference genome, proteins in this cluster had GGDEF (Gly-Gly-Asp-Glu-Phe) or EAL (Glu-Ala-Leu) domains, and sensor proteins for the two component regulatory system (Figure 4d). This same result was observed when the **Selected **or **Proteobacteria **reference genome sets were used. Previous studies have shown that quorum sensing and two component regulatory system are involved in biofilm formation [25,26]. Moreover, our experiments previously have shown that many of these previously uncharacterized GGDEF containing proteins can contribute to biofilm formation [27]. When the reference genome was changed to include genomes that shared a biofilm relevant phenotype (i.e. motility), the size of the cluster and the number of proteins within this cluster with different functional categories increased. The cluster still contained proteins with GGDEF or EAL domains but now included the sensors, amino acid biosynthesis proteins, and regulators that may contribute to the expression and regulation of overall biofilm phenotypes in *E. coli*. This result indicates that in at least some cases the reference selection can point out unique features of target organisms that would be missed had another reference genome been selected. Moreover, this result demonstrates that choice of reference genomes selection can also be used to identify Co-Conserved clusters of proteins that function in distinct pathways (regulators, cyclic-di-GMP metabolism, etc.) yet contribute to a common phenomenon (biofilms). This information is of substantial value to biological studies seeking to decipher complex phenotypes such as the biofilm phenotype examined here.

### Comparison to alternative methods

**Figure 5:**
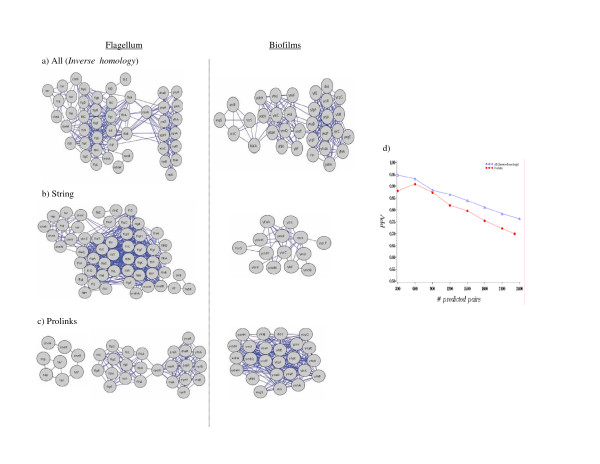
Comparison of our result to previous works using Co-Conservation of chemotaxis and flagellar proteins and GGDEF/EAL domains. a) Our refined method. The reference genome is **All **and *Inverse homology *applied to Pearson correlation coefficient used as scoring scheme. The confidence of r > 0.8 and threshold *E*-value 10^-5 ^was used. b) Predicted cluster for flagellar and GGDEF and EAL domains clusters by String (confidence 0.4) c) Predicted cluster for flagellar and GGDEF and EAL domains by Prolinks (confidence 0.6). d) The PPV for the top 2600 predicted pairs by Prolinks versus our method. Our method was *Inverse homology *applied to Pearson correlation coefficient r > 0.8 with *E*-value threshold 10^-5^.

We compared the performance of our method employing *Inverse homology *weighting to the performance of Prolinks and String databases both for predicting several well studied interaction networks and for overall PPV performance. Our scoring scheme weights the Phylogenetic profile vectors by taking into consideration the homology of the target genome versus the reference genome (*Inverse homology*) while the methods employed in the Prolinks and String databases do not consider the effect of the reference genome in their scoring scheme. It is of note that previously published data available from Prolinks showed that Prolinks had more linkages than other available sources at the time of implementation [8]. In that we have already demonstrated above the importance of reference genome selection, we used **All **as the reference genome set since almost all of the publicly accessible databases use all available bacterial genomes at the time of their implementation. In previous studies, we showed that there were several well known PPI pairs involved in flagellum or biofilm processes that were not identified in publicly available databases [28]. The bacteria flagellum process is a complex molecular system with multiple components required for functional motility, which extends from the cytoplasm to the cell exterior. While there are many common themes in flagellar protein control and assembly, there also appears to be variation among organisms. Some of the flagellar proteins were not identified in Prolinks [8] such as, three ring proteins (FlgH, FlgI, and FliF), some of the axle-like proteins (FliE, FlgB, FlgF, and FliD) that have been shown to physically interact with FlgB [29], and the stator motor proteins MotA and MotB. When *Inverse homology *is used in the determination of similarity, all of the above proteins were contained within the predicted protein interaction network (Figure 5a). In addition, proteins (FliT, FlgM, FlgN) that are species specific are not Co-Conserved as would be expected [30]. A similar result was observed to the biofilm protein interaction clusters described above, where several proteins that have known GGDEF and EAL domains were missed by Prolinks [8] and String [5] (Figure 5). Finally, direct comparison of the total number of predicted pairs indicated that using *Inverse homology *increased not only the number of predicted interaction pairs but also PPV over a large range of such predicted interactions (Figure 5d).

## Discussion and conclusion

The use of computational methods will continue to grow as more genomes are fully sequenced. Here we examined how differences in key aspects of the Phylogenetic profiles method affected predicted protein interaction networks. We specifically focused on aspects involving the selection of reference genomes and the measurement of similarity among protein Co-Conservation vector profiles.

Phylogenetic profiles method offer an alternative to strictly homology-based approaches. While homology-based methods can be effective for predicting the functions of remote homologs, these methods perform poorly as the evolutionary distance between homologous proteins increases. Even a sophisticated homology-based method fails to successfully assign functions to most of the proteins for a particular organism. Phylogenetic profiles methods on the other hand are not strictly based on homology and assign function to a protein based on the context of its interactions with other proteins within a cluster. We designed a new system that utilizes different features of this method and showed that these features affect the accuracy of predictions.

Pellegrini *et al. *introduced Phylogenetic profiles while using 16 fully sequenced organism [4]. Since more genomes have become available, the choice of reference genomes to use when constructing Phylogenetic profiles has become more important. Specifically, we noted that the number of unclassified proteins varied considerably depending on the reference set of genomes. This result both verified and extended previous results based on relatedness of reference genomes. We also showed that selection of all sequenced bacteria as a reference genome set may not produce the optimal PPV since the set of fully sequenced bacteria is biased towards pathogens and laboratory species (i.e. *E. coli*) among others. We showed that different sets of reference genomes produce substantially different results in the terms of the accuracy of predicted interactions (i.e. PPV), and that such results were relatively independent of the choice of *E*-value threshold employed to define homologs. This specific result demonstrates the need for flexibility in choosing among reference genomes when initiating Phylogenetic profiling based efforts for prediction protein interaction networks. One clear challenge here is that the selection of such reference genomes is not simple. Rather, this process requires knowledge of the relevant organisms, both in terms of their taxonomy and the specific phenotypes they express. To aid in this process, we introduced a new scoring scheme (*Inverse homology*) that considers the homology of the target and reference genomes, and thus places some emphasis on the evolutionary relationship of the relevant genomes. We showed that depending on the similarity metric used in combination with our *Inverse homology *scoring scheme, either the accuracy or the topology of the predicted protein interaction network was altered.

Our final studies were directed at understanding the extent to which these issues affected overall predictions and whether or not any observed differences provide new biological insights. We examined the topology of the network with various reference genomes and noted that when more closely related bacteria were selected, the clusters became larger with a higher degree of interconnectedness when compared to clusters derived from more distantly related reference genomes (Table 1, 2, 3). In general, small and medium sized clusters tend to contained proteins of known function in contrast to large clusters that contained proteins that function in distinct but coordinate processes. As such, Phylogenetic profiling-based approaches can benefit from flexibility in selecting and weighting of reference genomes as demonstrated here. We showed that such benefits do indeed generate unique biological insights. In particular, we showed that biofilm relevant protein interaction networks contained a broader range of relevant protein functions when reference genomes were selected based on a shared essential phenotype (motility) as compared to using all available genomes. We extended this result by comparing directly the use of our *Inverse homology *scoring scheme to the methods used by publicly available databases (i.e. Prolinks or String). The *Inverse homology *approach predicted known protein interactions in two separate biological processes (flagellum, biofilms) that were not predicted by existing methods.

Overall, we have presented an evaluation of several key criteria affecting the accuracy and topology of protein interaction networks predicted by Phylogenetic profiling methods. We have shown that the choice of reference genome is of key importance and provided guidance, both in terms of different evaluations and the report of a new similarity scoring scheme, for future efforts seeking to further improve computational methods for predicting protein interactions as well as to use such methods for developing new biological understanding.

## Methods

### Reference Genome Selection

At the time of our implementation (June 2006), 268 complete microbial genomes were available through the National Center for Biotechnology Information (NCBI) and were downloaded from their ftp site [31]. Phenotypic information such as motility and oxygen requirement was generated manually from available data on NCBI [32]. Several different reference genomes were used in our system and they were 1) **Proteobacteria **(130 bacteria), 2) **Low G+C Gram positive **bacteria (75 bacteria), 3)**High G+C Gram positive **bacteria (22 bacteria), 4) Selecting only one strain from those fully sequenced for each organism (**Selected **(75 bacteria)), 5) All the fully sequenced bacteria available on NCBI (**All **(268 bacteria)), 6) selecting based on oxygen requirement (**Aerobic **(91 bacteria), 7) **Anaerobic **(31 bacteria), and **Facultative **(107)), and 8) selecting based on Motility (**Motile **(104), and **None-Motile **(82)). In our evaluation we focused on a target genome *E. coli *K12 because a well curated dataset of protein functions is available [22] and substantial experimental data exists for this bacteria.

### Creating Phylogenetic profiles matrix

We performed pairwise one-against-all BLAST searches to identify all homologous target proteins in diverse reference organisms. For each protein i of the target organism, the BLAST *E*-value of the top scoring sequence alignment between protein i and all the proteins of each reference genome j was assign to E_ij_. The Phylogentic profile was constructed as a vector with elements P_ij_, where P_ij _= 1 if a homolog exists (E_ij _<*E*-value threshold) for same protein in genome j, otherwise P_ij _= 0. The number of the protein homologs varies depending upon our reference genome set and *E*-value threshold (Table 4). For example, when the reference genome set was **All **and threshold *E*-value of BLAST was 10^-5^, there were 289 proteins in *E. coli *K12 that appeared in more than 90% of the reference genome set and more than 53% of these were known essential proteins [33] compared to 211 proteins with a threshold of 10^-15^, 59% of these were known essentials.

**Table 4:** Proteins existing in more than 90% and less than 10% of genomes base on different set of reference genomes. (*E*-value 10^-5^)

Reference genome	No. proteins appearing in <10%	No. proteins appearing in > 90%
**Aerobic**	1,297	455
**All**	1,034	289
**High GC Gram positive**	1,912	333
**Low GC Gram negative**	1,899	723
**Motile**	745	104
**Proteobacteria**	614	499
**Selected**	1,198	446

Based on the assumption that highly conserved proteins (>90% of genomes evaluated) would be limited to a few functional categories and poorly conserved proteins are likely uncharacterized, we eliminated such proteins prior to measuring profile similarities (described below). To check this assumption, we characterized the discarded proteins based on COG classifications as shown in Additional file 2. The majority of proteins that appeared in more than 90% of the reference genomes were involved in translation, ribosomal structure and biogenesis, while the majority of proteins appearing in less than 10% of the reference genomes where unclassified.

### Generating weighted Phylogenetic profile vectors using Inverse homology

As an alternative to binary vectors, we also developed a weighting scheme which we refer to as *Inverse homology*. The *Inverse homology *was calculated by weighting the Phylogenetic profile vector by taking into consideration the homology of the target genome versus the reference genome. Given an *E*-value threshold, the homology H_i, j _between two genomes was calculated as the ratio of number of homologs of each reference organism j to the number of proteins in the target genome i. For each protein i the target, if there was a homolog to reference protein j (E_ij _<*E*-value threshold) then P_ij _= 1/(H_i, j_) otherwise P_ij _= 0. Calculating the P_ij _in this way, rather than using binary values as originally thought [4] or normalizing the *E*-values [7], incorporates genome homology information and accounts for phylogenetic relationships between genomes and improves estimates of profile similarities.

### Measuring profile similarities

Given a set of (weighted) Phylogenetic profiles, we can calculate the similarity between any pair of proteins using either Pearson correlation coefficient or Mutual Information. We describe each below.

#### Pearson correlation coefficient

Similarity between two protein vectors using Pearson correlation coefficient was calculated as [4,7,34]*f*_*X *_= (*I*/*N*), *f*_*Y *_= (*J*/*N*), and *f*_*Z *_= (*K*/*N*)

r=fZ−fXfY(fX−fX2)(fY−fY2)
 MathType@MTEF@5@5@+=feaafiart1ev1aaatCvAUfKttLearuWrP9MDH5MBPbIqV92AaeXatLxBI9gBaebbnrfifHhDYfgasaacH8akY=wiFfYdH8Gipec8Eeeu0xXdbba9frFj0=OqFfea0dXdd9vqai=hGuQ8kuc9pgc9s8qqaq=dirpe0xb9q8qiLsFr0=vr0=vr0dc8meaabaqaciaacaGaaeqabaqabeGadaaakeaacqWGYbGCcqGH9aqpdaWcaaqaaiabdAgaMnaaBaaaleaacqWGAbGwaeqaaOGaeyOeI0IaemOzay2aaSbaaSqaaiabdIfaybqabaGccqWGMbGzdaWgaaWcbaGaemywaKfabeaaaOqaamaakaaabaGaeiikaGIaemOzay2aaSbaaSqaaiabdIfaybqabaGccqGHsislcqWGMbGzdaqhaaWcbaGaemiwaGfabaGaeGOmaidaaOGaeiykaKIaeiikaGIaemOzay2aaSbaaSqaaiabdMfazbqabaGccqGHsislcqWGMbGzdaqhaaWcbaGaemywaKfabaGaeGOmaidaaOGaeiykaKcaleqaaaaaaaa@4AC1@

I is the sum of P_X, j _overall reference genome j, J is the sum of P_Y, j _over j. When the vectors are binary, K is the subset of genomes that contain homologs of both X and Y and N represent the total number of reference genomes. When the vectors are weighted by *Inverse homology*, K is the sum 1/H_i, j _over the subset of genomes that contain homologs of both X and Y and N is the sum of 1/H_i, j _over all j for target i.

#### Mutual Information

Several studies have used Mutual Information (MI) to assess protein functional linkage [4,7,34]. The Mutual Information MI (X, Y) is maximum when there is high covariation between two proteins and is defined by

MI(X,Y)=∑i,jpij(X,Y)log⁡pij(X|Y)pi(X)=∑i,jpij(X,Y)log⁡pij(X,Y)pi(X)pj(Y)
 MathType@MTEF@5@5@+=feaafiart1ev1aaatCvAUfKttLearuWrP9MDH5MBPbIqV92AaeXatLxBI9gBaebbnrfifHhDYfgasaacH8akY=wiFfYdH8Gipec8Eeeu0xXdbba9frFj0=OqFfea0dXdd9vqai=hGuQ8kuc9pgc9s8qqaq=dirpe0xb9q8qiLsFr0=vr0=vr0dc8meaabaqaciaacaGaaeqabaqabeGadaaakeaafaqadeGabaaabaGaemyta0KaemysaKKaeiikaGIaemiwaGLaeiilaWIaemywaKLaeiykaKIaeyypa0ZaaabuaeaacqWGWbaCdaWgaaWcbaGaemyAaKMaemOAaOgabeaaaeaacqWGPbqAcqGGSaalcqWGQbGAaeqaniabggHiLdGccqGGOaakcqWGybawcqGGSaalcqWGzbqwcqGGPaqkcyGGSbaBcqGGVbWBcqGGNbWzdaWcaaqaaiabdchaWnaaBaaaleaacqWGPbqAcqWGQbGAaeqaaOGaeiikaGIaemiwaGLaeiiFaWNaemywaKLaeiykaKcabaGaemiCaa3aaSbaaSqaaiabdMgaPbqabaGccqGGOaakcqWGybawcqGGPaqkaaaabaGaeyypa0ZaaabuaeaacqWGWbaCdaWgaaWcbaGaemyAaKMaemOAaOgabeaaaeaacqWGPbqAcqGGSaalcqWGQbGAaeqaniabggHiLdGccqGGOaakcqWGybawcqGGSaalcqWGzbqwcqGGPaqkcyGGSbaBcqGGVbWBcqGGNbWzdaWcaaqaaiabdchaWnaaBaaaleaacqWGPbqAcqWGQbGAaeqaaOGaeiikaGIaemiwaGLaeiilaWIaemywaKLaeiykaKcabaGaemiCaa3aaSbaaSqaaiabdMgaPbqabaGccqGGOaakcqWGybawcqGGPaqkcqWGWbaCdaWgaaWcbaGaemOAaOgabeaakiabcIcaOiabdMfazjabcMcaPaaaaaaaaa@818E@

Then to carry the sum using the quantities *f*_*X*_, *f*_*Y *_*and f*_*Z *_describe above, we used the equations below, as described by Wu *et. al *[4,7,34]

I1(X,Y)≡fZlog⁡fZfXfY
 MathType@MTEF@5@5@+=feaafiart1ev1aaatCvAUfKttLearuWrP9MDH5MBPbIqV92AaeXatLxBI9gBaebbnrfifHhDYfgasaacH8akY=wiFfYdH8Gipec8Eeeu0xXdbba9frFj0=OqFfea0dXdd9vqai=hGuQ8kuc9pgc9s8qqaq=dirpe0xb9q8qiLsFr0=vr0=vr0dc8meaabaqaciaacaGaaeqabaqabeGadaaakeaacqWGjbqsdaWgaaWcbaGaeGymaedabeaakiabcIcaOiabdIfayjabcYcaSiabdMfazjabcMcaPiabggMi6kabdAgaMnaaBaaaleaacqWGAbGwaeqaaOGagiiBaWMaei4Ba8Maei4zaC2aaSaaaeaacqWGMbGzdaWgaaWcbaGaemOwaOfabeaaaOqaaiabdAgaMnaaBaaaleaacqWGybawaeqaaOGaemOzay2aaSbaaSqaaiabdMfazbqabaaaaaaa@44FA@

I2(X,Y)≡(fX−fZ)log⁡(fX−fZ)fX(1−fY)
 MathType@MTEF@5@5@+=feaafiart1ev1aaatCvAUfKttLearuWrP9MDH5MBPbIqV92AaeXatLxBI9gBaebbnrfifHhDYfgasaacH8akY=wiFfYdH8Gipec8Eeeu0xXdbba9frFj0=OqFfea0dXdd9vqai=hGuQ8kuc9pgc9s8qqaq=dirpe0xb9q8qiLsFr0=vr0=vr0dc8meaabaqaciaacaGaaeqabaqabeGadaaakeaacqWGjbqsdaWgaaWcbaGaeGOmaidabeaakiabcIcaOiabdIfayjabcYcaSiabdMfazjabcMcaPiabggMi6kabcIcaOiabdAgaMnaaBaaaleaacqWGybawaeqaaOGaeyOeI0IaemOzay2aaSbaaSqaaiabdQfaAbqabaGccqGGPaqkcyGGSbaBcqGGVbWBcqGGNbWzdaWcaaqaaiabcIcaOiabdAgaMnaaBaaaleaacqWGybawaeqaaOGaeyOeI0IaemOzay2aaSbaaSqaaiabdQfaAbqabaGccqGGPaqkaeaacqWGMbGzdaWgaaWcbaGaemiwaGfabeaakiabcIcaOiabigdaXiabgkHiTiabdAgaMnaaBaaaleaacqWGzbqwaeqaaOGaeiykaKcaaaaa@535B@

I3(X,Y)≡(fY−fZ)log⁡(fY−fZ)fY(1−fX)
 MathType@MTEF@5@5@+=feaafiart1ev1aaatCvAUfKttLearuWrP9MDH5MBPbIqV92AaeXatLxBI9gBaebbnrfifHhDYfgasaacH8akY=wiFfYdH8Gipec8Eeeu0xXdbba9frFj0=OqFfea0dXdd9vqai=hGuQ8kuc9pgc9s8qqaq=dirpe0xb9q8qiLsFr0=vr0=vr0dc8meaabaqaciaacaGaaeqabaqabeGadaaakeaacqWGjbqsdaWgaaWcbaGaeG4mamdabeaakiabcIcaOiabdIfayjabcYcaSiabdMfazjabcMcaPiabggMi6kabcIcaOiabdAgaMnaaBaaaleaacqWGzbqwaeqaaOGaeyOeI0IaemOzay2aaSbaaSqaaiabdQfaAbqabaGccqGGPaqkcyGGSbaBcqGGVbWBcqGGNbWzdaWcaaqaaiabcIcaOiabdAgaMnaaBaaaleaacqWGzbqwaeqaaOGaeyOeI0IaemOzay2aaSbaaSqaaiabdQfaAbqabaGccqGGPaqkaeaacqWGMbGzdaWgaaWcbaGaemywaKfabeaakiabcIcaOiabigdaXiabgkHiTiabdAgaMnaaBaaaleaacqWGybawaeqaaOGaeiykaKcaaaaa@5361@

I4(X,Y)≡(1−fX−fY+fZ)log⁡(1−fX−fY+fZ)(1−fX)(1−fY)
 MathType@MTEF@5@5@+=feaafiart1ev1aaatCvAUfKttLearuWrP9MDH5MBPbIqV92AaeXatLxBI9gBaebbnrfifHhDYfgasaacH8akY=wiFfYdH8Gipec8Eeeu0xXdbba9frFj0=OqFfea0dXdd9vqai=hGuQ8kuc9pgc9s8qqaq=dirpe0xb9q8qiLsFr0=vr0=vr0dc8meaabaqaciaacaGaaeqabaqabeGadaaakeaacqWGjbqsdaWgaaWcbaGaeGinaqdabeaakiabcIcaOiabdIfayjabcYcaSiabdMfazjabcMcaPiabggMi6kabcIcaOiabigdaXiabgkHiTiabdAgaMnaaBaaaleaacqWGybawaeqaaOGaeyOeI0IaemOzay2aaSbaaSqaaiabdMfazbqabaGccqGHRaWkcqWGMbGzdaWgaaWcbaGaemOwaOfabeaakiabcMcaPiGbcYgaSjabc+gaVjabcEgaNnaalaaabaGaeiikaGIaeGymaeJaeyOeI0IaemOzay2aaSbaaSqaaiabdIfaybqabaGccqGHsislcqWGMbGzdaWgaaWcbaGaemywaKfabeaakiabgUcaRiabdAgaMnaaBaaaleaacqWGAbGwaeqaaOGaeiykaKcabaGaeiikaGIaeGymaeJaeyOeI0IaemOzay2aaSbaaSqaaiabdIfaybqabaGccqGGPaqkcqGGOaakcqaIXaqmcqGHsislcqWGMbGzdaWgaaWcbaGaemywaKfabeaakiabcMcaPaaaaaa@61F8@

Then

*I*(*X*, *Y*) = *I*_1_(*X*, *Y*) + *I*_2_(*X*, *Y*) + *I*_3_(*X*, *Y*) + *I*_4_(*X*, *Y*).

Note: we use log_2 _when the vectors are binary and log_e _when the vectors are weighted by *Inverse homology*.

### Generating the protein-protein interaction network

Networks were created and presented as graphs in which each protein is represented as a node and an interaction between proteins is represented by an edge. An edge exists between a pair of proteins whose Phylogenetic profiles similarity score exceed a given threshold. For separation of connected components of the network and building the clusters of proteins, breadth-first search (BFS) graph algorithms were used. The target *E. coli *K12 genome was analyzed, and the number of assigned pairs is shown in Table 1 and Table 2. Network graphs were visualized using Cytoscape [35], an open-source, platform-independent environment software. The lengths of the lines connecting proteins hold no meaning and vary to facilitate viewing of the network.

### *E*-value threshold

We examined whether changing the BLASTP threshold *E*-value would affect the accuracy and performance of the Co-Conservation method. For determination and optimization of the *E*-value for each organism, four *E*-values were applied to determine when a homologous protein was present or absent, 1 × 10^-15^, 1 × 10^-10^, 1 × 10^-5^, and 1 × 10^-3^. An *E*-value was considered optimal if it had the maximum number of correctly linked proteins, as ranked by the selected scoring scheme. A correct linkage was defined by two proteins sharing the same biological process. For evaluation purposes we used on eight different types of reference genomes. Therefore, 32 combinations of different reference organism sets and various *E*-values thresholds were formed and were evaluated using COG functional categories and EcoCyc.

### Analyzing the topology of the network

The degree of a node in a graph is the number of edges connected to that node and proteins that are joined by an edge are said to be adjacent. A neighbor of a protein i is a protein adjacent to i. The clustering coefficient C indicates the degree to which k neighbors of a particular node are connected to each other. Let k_i _be the number of neighbors of node i and k_i-1 _be the number of nodes connected to neighbors of i. The clustering coefficient of node i is given as

C_i _= 2 n_i_/k_i _* (k_i-1_)

where n_i _is the number of edges that exist between i, its neighbors and their neighbors [23]. Then the average clustering coefficient was calculated by averaging C over all nodes i.

### Comparison of predicted protein interaction to published data and available resources

In order to measure the performance and reliability of our method over previous methods, we compared the number of interacting proteins, the number of predicted unknown proteins and the functional similarity of proteins sharing a protein-protein interaction.

We evaluated the predicted protein-protein interaction data of *E. coli *K12, based on Clusters of Orthologous Groups of proteins (COG) in NCBI [36] e.g. for *E. coli *K12 [31]), biological pathway information in KEGG orthology (KO) [37] five broad functional categories (Metabolism, Genetic Information Processing, Environmental Information Processing, Cellular Processes, and Human Diseases), TIGR [38] functional role category (18 different functional role categories), and protein complexes, pathway, operons, regulator (pairs of gene A and B, where the product of gene A is the a component of transcription factor that regulates gene B), and paralogous groups in EcoCyc. EcoCyc (June 2006) data were downloaded from [22], we extracted all information related to protein complexes, pathway, operons, regulators, and paralogous groups from EcoCyc.

To find out whether the selection of bacteria makes a difference and what the optimal way to select the bacteria is, we compared the performance of PPV using different types of reference genomes. The predicted pairs where at least one protein was unclassified were removed from analysis. In addition the interactions that involved proteins that are classified as "General function" in COG function categories were considered unclassified in COG. The functional similarity of a protein interaction dataset for COG was a true positive (TP) where the pair had the same functional category and a false positive (FP) where they belong to different functional categories. Similarly, for the EcoCyc database, proteins that appear in the same complex, pathway, operons, homologs and paralogous group are presumed to be true positives, while the other classified pairs were false positives. Sensitivity and Specificity are good predictive values but since the negative data set can not be defined, we used the Positive Predictive Value (PPV). The PPV was calculated as PPV = TP/(TP+FP). Finally, we compared our result to previous works such as String [5] and Prolinks [8] databases. Though String and Prolinks employ a variety of methods for predicting interactions, only those interactions based solely on Phylogenetic profiles were extracted. Prolinks makes available all interactions together with a confidence score for each interaction. We compared the top 2,600 interactions obtained by *Inverse homology *against the top 2,600 interactions from Prolinks by calculating the PPV using the EcoCyc database for *E. coli *K12 (Figure 5d). We also analyzed several clusters involving well known processes (i.e. flagellum, chemotaxis, and biofilm proteins), as described in the Results section in detail, against interactions from String and Prolinks.

## Authors' contributions

AK, LH and RTG design the study. AK implemented the methods and analyzed the data. The manuscript was written by AK and edited by RTG and LH. RTG oversaw all biological aspects of the work and LH supervised the computational aspect.

## Supplementary Material

Additional file 1 Comparison of different combinations of reference genomes set and *E*-value thresholds. a) E^-3 ^b) E^-5 ^c) E^-10 ^d) E^-15^. Positive Predicted Value (PPV) was calculated using EcoCyc functional category as described in Materials and Methods section. Click here for file

Additional file 2 Functional classification of proteins appearing in more than 90% and less than 10% of organisms using COG. a) Proteins that appear in more than 90% of reference genomes (Reference genome is **All**). b) Proteins that appear in less than 10% of reference genomes (Reference genome is **All**). c) Proteins that appear in more than 90% of reference genomes (Reference genome is **Motile**). d) Proteins that appear on less than 10% of genomes reference genomes (Reference genome is **Motile**). *E*-value threshold is 10^-5^. COG functional categories are: Translation, ribosomal structure and biogenesis (**J**) ; Transcription (**K**); DNA replication, recombination and repair (**L**); Cell division and chromosome partitioning (**D**) ; Posttranslational modification, protein turnover, chaperones (**O**); Cell envelope biogenesis, outer membrane (**M**); Cell motility and secretion (**N**); Inorganic ion transport and metabolism (**P**); Signal transduction mechanism (**T**), Energy production and conversion (**C**); Carbohydrate transport and metabolism (**G**), Amino acid transport and metabolism (**E)**; Nucleotide transport and metabolism (**F**); Coenzyme metabolism **(I); **Lipid metabolism (**H**); Secondary metabolites biosynthesis, transport and catabolism (**Q)**; General function prediction only (**R**); Function unknown (**S**); Not classified (**-**); Intracellular trafficking, secretion, and vesicular transport (**U**), Defense mechanisms (**V**); RNA processing and modification (**A**). Click here for file
