# Supplementary figures and images for: Investigation of factors affecting prediction of protein-protein interaction networks by phylogenetic profiling
Source: BMC Genomics. 2007 Oct 29;8:393. doi: 10.1186/1471-2164-8-393 (PMC2204017; doi:10.1186/1471-2164-8-393)

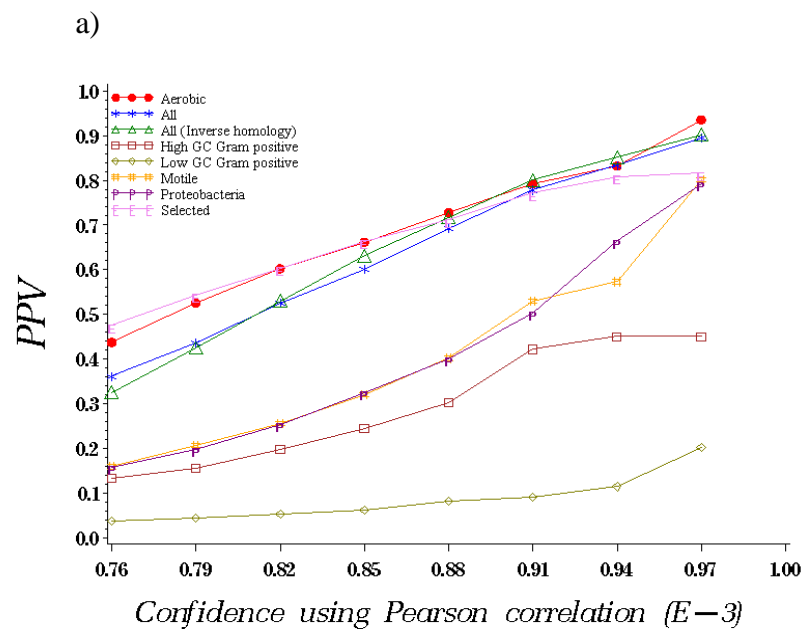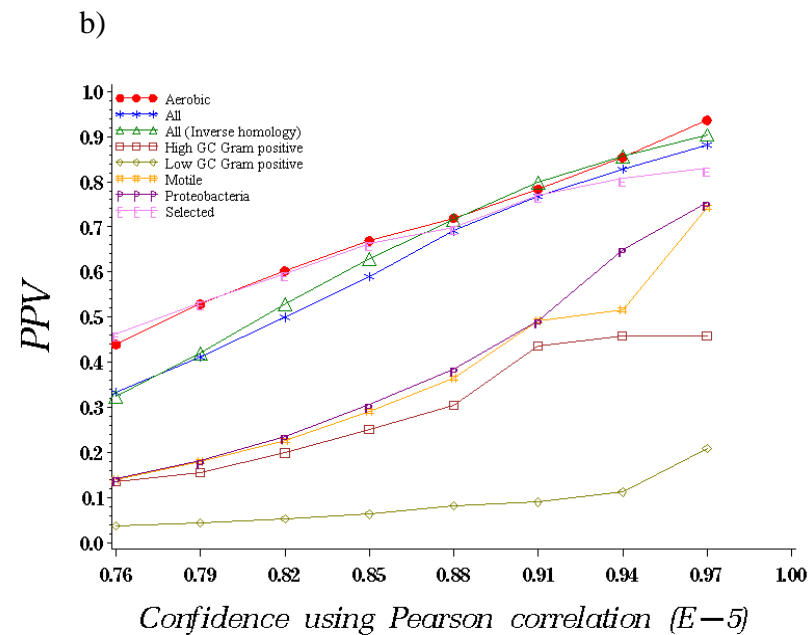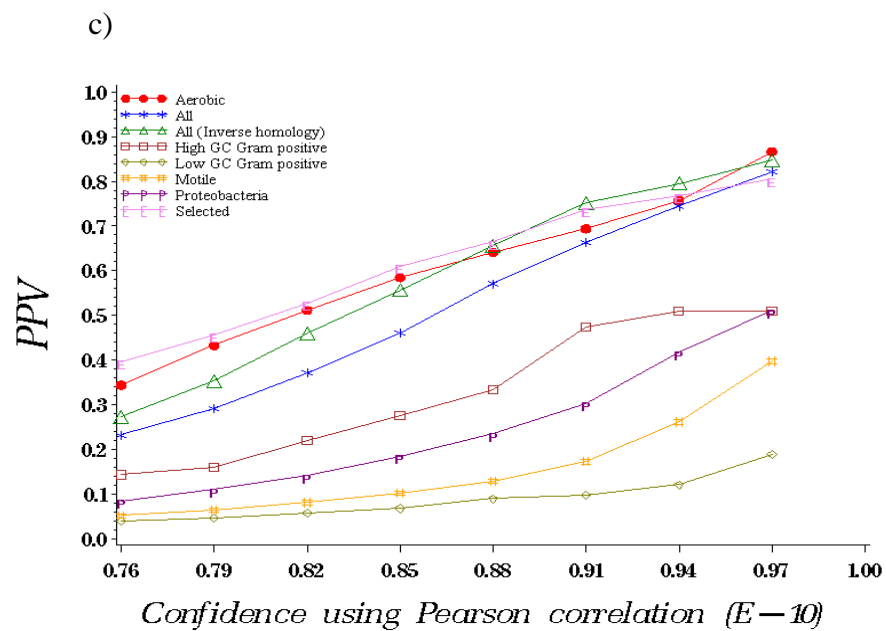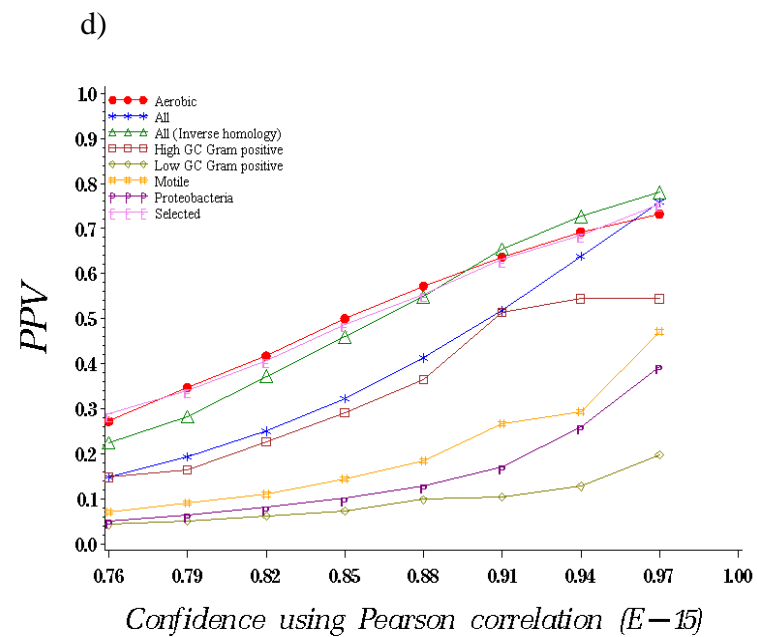

Supplement: Additional file 1 — Comparison of different combinations of reference genomes set and E-value thresholds. a) E-3 b) E-5 c) E-10 d) E-15. Positive Predicted Value (PPV) was calculated using EcoCyc functional category as described in Materials and Methods section. [file 1471-2164-8-393-S1.pdf]

a)

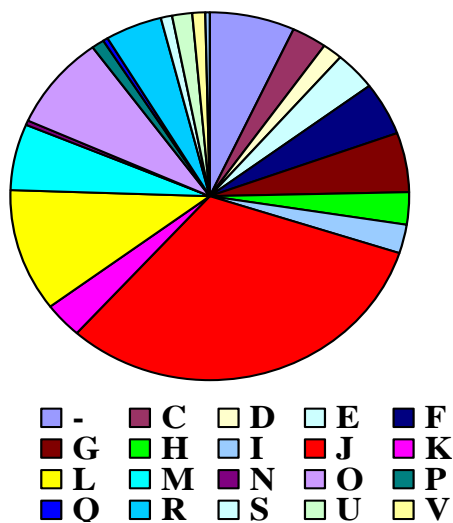

b)

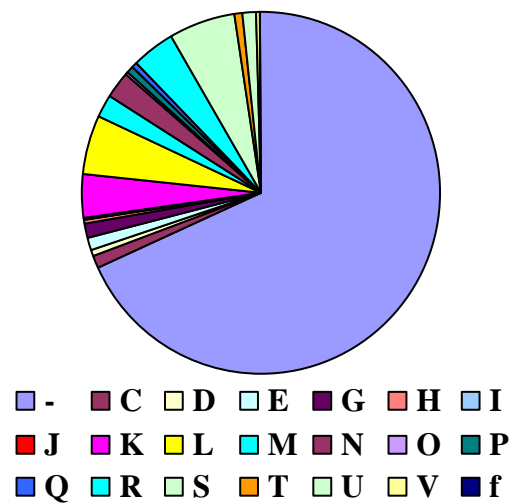

c)

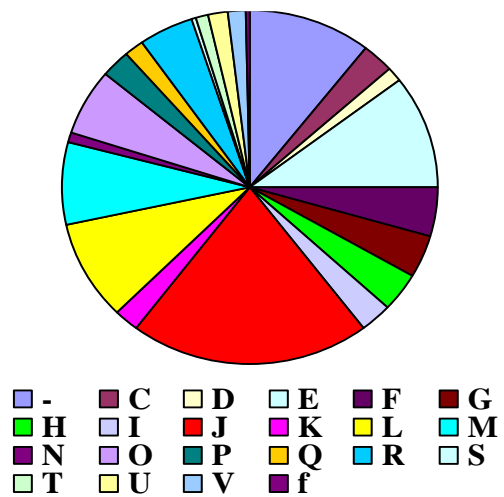

d)

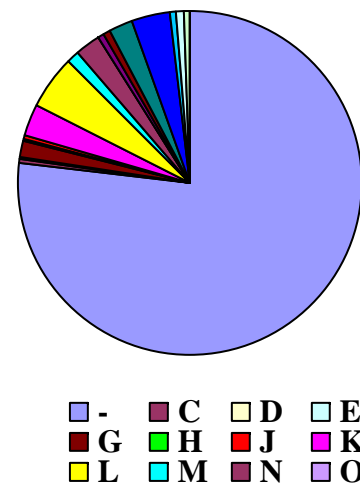

Supplement: Additional file 2 — Functional classification of proteins appearing in more than 90% and less than 10% of organisms using COG. a) Proteins that appear in more than 90% of reference genomes (Reference genome is All). b) Proteins that appear in less than 10% of reference genomes (Reference genome is All). c) Proteins that appear in more than 90% of reference genomes (Reference genome is Motile). d) Proteins that appear on less than 10% of genomes reference genomes (Reference genome is Motile). E-value threshold is 10-5. COG functional categories are: Translation, ribosomal structure and biogenesis (J) ; Transcription (K); DNA replication, recombination and repair (L); Cell division and chromosome partitioning (D) ; Posttranslational modification, protein turnover, chaperones (O); Cell envelope biogenesis, outer membrane (M); Cell motility and secretion (N); Inorganic ion transport and metabolism (P); Signal transduction mechanism (T), Energy production and conversion (C); Carbohydrate transport and metabolism (G), Amino acid transport and metabolism (E); Nucleotide transport and metabolism (F); Coenzyme metabolism (I); Lipid metabolism (H); Secondary metabolites biosynthesis, transport and catabolism (Q); General function prediction only (R); Function unknown (S); Not classified (-); Intracellular trafficking, secretion, and vesicular transport (U), Defense mechanisms (V); RNA processing and modification (A). [file 1471-2164-8-393-S2.pdf]
